# A New Method for the Restoration of Respiration Lost under Chloroform

**Published:** 1887-04

**Authors:** 


### A New Method for the Restoration of Respiration Lost under Chloroform.

Dr. R. Milne Murray describes a new method for the restoration of respiration lost under chloroform, which he terms "perflation," as follows:

"Disconnecting the rubber tubing, I take the end of the branch with my finger, and make one or two aspirations of the lungs, compressing the chest gently at the same time. This removes a considerable quantity of vapor from the upper passages. Then, opening the branch, I make a series of deep inspirations.

"The air rushes in by the branch, and no doubt the greater part passes into the mouth; yet some of it enters the lungs, and a current is thus established by which a very large quantity of the chloroform is rapidly expelled, as can be proved by the taste of the air coming through the tube. After two or three such inspirations, the taste of the vaper becomes fainter, and as soon as this is noticed, I reverse the process, now blowing air into the tube, with force just sufficient to cause the chest-wall to move in the slightest possible degree—the branch tube being open all the time. Generally, after one or two such perflations, the heart shows signs of vigorous action, and shortly thereafter breathing commences, and continues in a perfectly natural manner. Should it not return so rapidly, and after I am assured by the absence of taste or smell in the expired air that the chloroform has been almost entirely removed, then I close the branch tube, and commence gentle inflation of the lungs in the ordinary way.

"Speaking broadly, as regards the difficulty of resuscitation as indicated by the time required to effect it, I have observed that *the time required to restore respiration varies inversely as the concentration of the dose, and directly as the time required to stop respiration*—in other words, the more concentrated the dose the easier was the reanimation, and the longer respiration continued under the action of the vapor the more difficult was the reanimation."—*Edinburgh Med. Jour.*

